# Botulinum Neurotoxin Type A Combined with Platelet-Rich Plasma Promotes Hair Follicle Growth and Regeneration via STMN1 Modulation and Wnt/β-Catenin Pathway Activation

**DOI:** 10.4014/jmb.2507.07033

**Published:** 2025-11-26

**Authors:** Jiangxia Li, Zhenyu Jia, Yin Zhou, Liang Gao, Yan Ma

**Affiliations:** 1 Heji Hospital Affiliated to Changzhi Medical College, P.R. China; 2 Department of Dermatology and Venereology, the Second Hospital of Shanxi Medical University, P.R. China

**Keywords:** BoNT/A, PRP, hair follicle growth and reconstruction, STMN1, Wnt/β-catenin signaling pathway

## Abstract

There is currently limited knowledge about the combined effect of Botulinum toxin type A (BoNT/A) and Platelet-rich plasma (PRP) on hair follicle growth and reconstruction. This study aimed to investigate the impact of BoNT/A in conjunction with PRP on hair follicles and explore its underlying mechanisms. The levels of alkaline phosphatase (ALP), fibroblast growth factor (FGF), platelet-derived growth factor (PDGF), vascular endothelial growth factor (VEGF), and versican levels were assessed to determine their influence on hair follicle growth. Hair follicle quantification was performed, while β-catenin and Ki-67 expression levels were analyzed. The concentrations of interleukin-6 (IL-6) and tumor necrosis factor-α (TNF-α) were measured as inflammatory biomarkers. Western blot analysis measured stathmin 1 (STMN1) expression and quantified key Wnt/β-catenin signaling pathway components. The findings demonstrated that the conjunction of BoNT/A and PRP effectively enhanced hair follicle growth. It promoted the upregulation of ALP, FGF, PDGF, VEGF, and versican, increased hair follicle number, upregulated ALP levels, and enhanced the expression of β-catenin and Ki-67. It also markedly decreased the concentrations of IL-6 and TNF-α, potentially inhibiting hair follicle miniaturization by mitigating inflammation. The combined treatment promotes hair follicle growth by activating the Wnt/β-catenin pathway through STMN1 upregulation. BoNT/A in conjunction with PRP facilitates hair follicle growth and reconstruction through STMN1 protein modulation and Wnt/β-catenin pathway activation.

## Introduction

Androgenetic alopecia (AGA), also known as seborrheic alopecia, is the most common form of hair loss, comprising nearly 90% of all cases [1]. AGA is a non-scarring form of hair loss marked by progressive follicular miniaturization due to shortened anagen phase duration [2, 3]. This process involves the replacement of thick, pigmented terminal hairs with fine, unpigmented vellus-like hairs and ultimately results in severe follicular atrophy and hair loss. AGA is easy to diagnose but difficult to treat. Early diagnosis and treatment can slow down the progression of hair loss. Currently, the treatment methods for AGA include: topical medications, oral medications, hair transplantation, laser therapy, and microneedle therapy, etc. [4-7]. Although several treatment options for AGA are currently available, monotherapy often yields suboptimal results and requires a prolonged treatment duration, which may increase the risk of adverse effects and compromise patient adherence. Therefore, investigating safe and effective combination therapies holds significant promise for enhancing the therapeutic outcomes of androgenetic alopecia.

Recent advances in medical technology have spurred growing research into platelet-rich plasma (PRP) applications for AGA, suggesting that PRP has the potential to serve as an effective and reliable adjunctive therapy for AGA [8, 9]. PRP is a concentrated preparation of platelets, with platelet concentrations reaching 4 to 8 times that of whole blood [10]. Upon activation, platelets release significant amounts of various growth factors, including epidermal growth factor (EGF), vascular endothelial growth factor (VEGF), and fibroblast growth factor (FGF), etc. [11]. Activated PRP exerts its therapeutic effects on androgenetic alopecia by prolonging the anagen phase and suppressing hair follicle cell apoptosis through growth factor secretion [12]. Studies have demonstrated that PRP exerts a positive influence on hair follicles and the hair growth cycle [13]. Although the exact mechanism of action of PRP remains unclear, it is widely acknowledged that PRP represents a promising therapeutic option for AGA.

Botulinum toxin (BoNT) is widely recognized for its dual applications in cosmetic procedures and the treatment of neurological and dermatological disorders [14]. Despite the clinical availability of multiple botulinum toxin subtypes, only types A and B have received regulatory approval for therapeutic applications [15]. In individuals genetically predisposed to AGA, increased dihydrotestosterone (DHT) production triggers excessive androgen receptor activation in hair follicles, which induces follicular miniaturization. This process progressively thins terminal hairs in androgen-sensitive scalp areas, ultimately causing their loss [16, 17]. Preliminary *in vitro* and animal studies indicate that BoNT may enhance hair growth in AGA by improving scalp blood flow and oxygenation while counteracting DHT-induced follicular miniaturization through TGF-β1 cytokine suppression [18, 19]. The relatively high cost of BoNT presents a challenge. To enhance cost-effectiveness, one potential approach could be combining BoNT with other "skin boosters," such as PRP. Further studies should evaluate the combined efficacy of these treatments administered alongside BoNT [14]. Wnt/β-catenin signaling critically regulates embryonic hair follicle morphogenesis, development, and regeneration throughout the hair cycle [20]. Research indicates that the microtubule-associated protein STMN1 can activate the Wnt/β-catenin signaling pathway, thereby promoting osteogenic differentiation [21]. Furthermore, studies have confirmed that STMN1 serves as a key factor in facilitating hair follicle regeneration [22].

Thus, in this study, this project aims to clarify the therapeutic value of combining BoNT/A with PRP in AGA treatment and to elucidate the potential pathways and targets involved in this combination therapy. The findings will provide more comprehensive experimental evidence for the application of BoNT/A combined with PRP in AGA treatment, thereby contributing significantly to both academic research and clinical practice.

## Materials and Methods

### Preparation and Activation of PRP Releasate (PRP-r)

After anesthetizing C57/BL6J mice, blood was collected by abdominal-aorta exsanguination via laparotomy, and 5 ml of anticoagulant was immediately added to prevent coagulation. PRP was isolated through a two-step centrifugation protocol. Initial centrifugation of the anticoagulated whole blood at 300 ×*g* for 10 min separated plasma from cellular constituents. The plasma supernatant, located more than 2 mm above the interface, was carefully transferred to another centrifuge tube. Second, the supernatant (PRP-releasate) was centrifuged at 1,900 ×*g* for 10 min to concentrate the platelets further. A total of 10 ml of PRP-releasate was obtained (PRP-releasate was a mixed preparation from the blood of 20 mice). Subsequently, the PRP-releasate was mixed with an activator solution containing 10% CaCl_2_ and 1000 U of bovine thrombin at a ratio of 10:1 and incubated at 4°C for 24 h to allow platelets to contract fully. After incubation, the sample was centrifuged again, and the supernatant was collected. PRP-releasate was prepared, sealed with parafilm, labeled, and stored at 4°C.

### Cell Culture

Dermal Papilla Cells (DPCs) (MIC-iCell-s015, iCell, China) were cultured in ICell Primary Fibroblast Culture System (PriMed-iCell-003, iCell, China). BoNT/A (National Drug Approval No. S10970037, Lanzhou Institute of Biological Products Co., Ltd, China) was applied to cells. DPCs were randomly divided into the following groups: Control group, BoNT/A group, PRP group, BoNT/A+PRP group, BoNT/A+PRP+siRNA-NC group (transfected with control small interfering RNA sequence, treated with BoNT/A and PRP), and BoNT/A+PRP+ siRNA-STMN1 group (transfected with an STMN1 small interfering RNA sequence, treated with BoNT/A and PRP). We prepared the riboFECT CP transfection complex. Adjusted DPCs density to 1×10^5^ cells/ml and seeded them in six-well plates. Once the cells had adhered, the transfection complex and gently mixed and after transfection, the transfection efficiency was assessed using Western blot.

### Enzyme-Linked Immunosorbent Assay (ELISA)

The skin tissues collected from the back hair follicles and the DPCs supernatant were used for subsequent analysis. The ALP ELISA KIT (A059-2-1, China), Mouse FGF ELISA KIT (ZC-38040, Zhuocai Biotechnology, China), Mouse PDGF ELISA KIT (ZC-38897, Zhuocai Biotechnology), Mouse VEGF ELISA KIT (ZC-38844, Zhuocai Biotechnology), Mouse IL-6 ELISA KIT (ZC-37988, Zhuocai Biotechnology), and Mouse TNF-α ELISA KIT (ZC-39024) expressions were detected according to the ELISA kit instructions.

### Immunofluorescence Staining

The DPC slides before treatment with the membrane-permeabilizing solution. Following a 10-min incubation at room temperature, the slides underwent another PBS wash. Then, the bovine serum albumin (BSA) (GC305010, Servicebio, China) was added, and the slides were incubated at room temperature for 20 min. After removing the blocking solution, we incubated the slides overnight at 4°C with the following primary antibodies: anti-versican (1:100, A19655, ABclonal, China), anti-β-catenin (1:200, 51067-2-AP, Proteintech, China), and anti-STMN1 (1:200, bs-1902R, Bioss, China). Then, FITC-labeled goat anti-rabbit (1:100, GB23303, Servicebio) was added. The DAPI (G1012, Servicebio) staining solution was added, and the slides were incubated at room temperature in the dark for 10 min. Finally, after washing with PBS, the slides were mounted. The images were taken with an OlyVIA microscopic camera system (Olympus, Japan).[Table T1]

### Real-Time Fluorescent Quantitative Polymerase Chain Reaction (RT-qPCR) Assay

RNA was isolated from the skin tissues collected from the back hair follicles separately. The isolated RNA was then reverse-transcribed into cDNA using the Reverse Transcription Kit (2690S, TaKaRa, Japan). Real-time fluorescence quantitative analysis was conducted. The thermal cycling conditions for qPCR assays: initial denaturation, 95°C, 30 sec; 45 cycles, 95°C, 5 sec; annealing, 55°C, 30 sec; extension, 72°C, 30 sec. SMNT1 mRNA expression level was calculated using the comparative CT method (2^-ddCT^ method).

### Cell Counting Kit-8 (CCK-8) Assay

DPCs in the logarithmic growth phase were collected through trypsinization and plated into 96-well plates. After the drug had acted for 48 h, the supernatant was aspirated and discarded. Each well received 110 μl of CCK-8 solution, followed by an additional 2 h incubation period. Subsequently, the absorbance was measured at a wavelength of 450 nanometers.

### Animal Grouping and Intervention

Fifteen male SPF-grade BALB/C nude mice (4 weeks old) were obtained from GemPharmatech Co., Ltd. and twelve male SPF-grade C57BL/6J mice (6~8 weeks old) were obtained from SPF (Beijing) Biotechnology Co., Ltd. All rats had free access to food and water, and housing conditions, including ventilation, complied with institutional animal care standards approved by the Animal Experiment Ethics Committee of Changzhi Medical College (No. DW2024191). After the adaptive feeding period, fifteen BALB/C nude mice were randomly allocated into three groups (*n* = 5): the Control group: subcutaneous injection of DMEM culture medium was performed around the transplanted hair follicles, with 10 μl injected per hair follicle; the DPCs group: subcutaneous injection of DPCs cell suspension (1 × 10^7^ cells/ml) was administered around the transplanted hair follicles, with 10 μl injected per hair follicle; the DPCs+BoNT/A+PRP: subcutaneous injection of DPCs cell suspension (1 × 10^7^ cells/ml), which had been cultured with 12.5 U/ml BoNT/A and 10% PRP, was performed around the transplanted hair follicles, with 10 μl injected per hair follicle.

### Establishment of the Hair Follicle Transplantation Model

(1) Extraction of whisker hair follicles from C57BL/6J mice: Following the completion of the adaptive feeding period, male C57BL/6J mice were euthanized. The whisker pads were excised and disinfected by immersion in 75% alcohol for 1 min, followed by three washes with PBS. Under a stereomicroscope (SMZ-168, Motic, China), subcutaneous fascia and adipose tissue were carefully removed using microscissors. Hair follicles in the anagen phase were identified and gently extracted with microforceps. The extracted hair follicles were collected in a sterile culture dish and washed three times with PBS. Hair follicles of consistent thickness were then selected and transferred to a new sterile culture dish for subsequent complete hair follicle transplantation.

(2) Transplantation of C57BL/6J mouse hair follicles into BALB/C nude mice: Nude mice were subjected to gas anesthesia. Upon successful induction of anesthesia, the dorsal skin was disinfected three times with 75% alcohol. With the midline of the spine as a reference, five hair follicles were transplanted sequentially from top to bottom on both sides of the back. A 17G syringe needle was used to create holes in the skin at an approximate angle of 10°relative to the skin surface until reaching the subcutaneous layer. Five holes were created on each side. The hair follicles were carefully held with microforceps in the right hand, while the pre-made holes were gently expanded using the needle held in the left hand. The hair follicles were then delicately inserted into the subcutaneous layer of the nude mouse. Special care was taken to minimize potential damage to the hair follicles during transplantation. Following transplantation, the exposed hair shafts were trimmed as short as possible to reduce the risk of hair follicle dislodgement due to friction caused by the nude mouse's movements. Photographs were captured on the first postoperative day and subsequently at 7-day intervals for a total duration of 28 days to document the growth dynamics of the transplanted hair follicles.

### Hematoxylin-Eosin (H&E) Staining

Skin tissues from BALB/C nude mice were fixed in 4% paraformaldehyde, sequentially dehydrated in ethanol, cleared in xylene for 40 min, embedded in paraffin, sectioned at 4–5 μm thickness, and stained with hematoxylin and eosin. The sections were observed using a Slide scanning imaging system (SQS-600P, Shenzhen Shengqiang Technology Co., Ltd.), and images were captured with the ImageViewer image analysis software. The mouse skin tissue was manually counted under a microscope to count the skin hair follicles.

### Immunohistochemical Experiment

The skin tissues of BALB/C nude mice were fixed in 4% paraformaldehyde, dehydrated in ethanol step by step. After cooling, the sections were washed with PBS. Place the sections in 3% hydrogen peroxide at room temperature in the dark for incubation, then add BSA and incubate at room temperature for 20 min. Afterward, the sections were incubated overnight with primary antibodies of β-catenin (1:500, ab32572, Abcam, China) and Ki67 (1:400, HA721115, HUABIO, China) followed by PBS washing. After incubation with a secondary antibody at 37°C for 30 min, tissue sections were stained with DAB and counterstained with hematoxylin. Following dehydration and mounting, images were acquired using a digital microscope (BA400Digital, Motic, China) and analyzed with specialized software.

### Western Blot Analysis

Total protein from DPCs was extracted using lysis buffer, then lysed on ice for 10 min to collect cells. Cells were centrifuged at 12,000 rpm at 4°C for 10 min to obtain supernatant. Subsequently, the cell supernatant was collected and the protein concentration was determined. After denaturation, the proteins were separated, transferred to a membrane, and blocked for 1 h. The following primary antibodies were incubated overnight at 4°C: STMN1 antibody (1:1000, 82559-1-RR, Proteintech, China), β-catenin antibody (1:500, A11512, ABclonal, China), GSK3β antibody (1:1000, A6164, ABclonal), versican antibody (1:1000, A19655, ABclonal, China), WNT10B antibody (1:2000, A16717, ABclonal), LaminB antibody (1:50000, 12987-1-AP, Proteintech), and β-actin antibody (1:50000, AC026, ABclonal). The membrane followed by incubation with HRP-labeled secondary antibody for 1 h. After three additional TBST washes, chemiluminescent solution was applied dropwise for development.

### Statistical Analysis

Data were analyzed with GraphPad Prism 9.0 and presented as mean ($\bar{X}$) ± standard deviation (SD). Comparisons among multiple groups were conducted using one-way ANOVA, followed by Tukey’s post hoc test. Between-group differences were assessed using t-tests. Statistical significance was set at *P* < 0.05.

## Results

### BoNT/A and PRP Promote Hair Follicle Growth

**Fig. 1:**
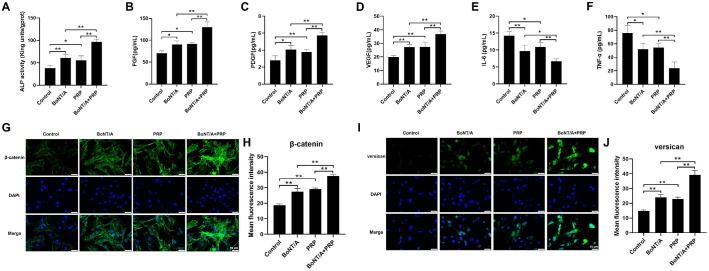
Analysis of the effects of BoNT/A and PRP on hair follicle growth. (**A-F**) The levels of ALP, FGF, PDGF, VEGF, IL-6, and TNF-α in DPCs. B: Immunofluorescence staining was used to detect the expression levels of β-catenin and versican (20X). The data are expressed as the mean ± SD. *P < 0.05, **P < 0.01.

To investigate the effects of BoNT/A and PRP on hair follicle growth, ELISA quantified growth-related factor concentrations (Fig. 1A-1F). The results demonstrated that, compared with the Control group, the levels of ALP, FGF, PDGF, and VEGF were significantly elevated in both the BoNT/A and PRP groups, while IL-6 and TNF-α levels decreased substantially (*P* < 0.05, *P* < 0.01). Furthermore, the combination treatment of BoNT/A and PRP exhibited superior efficacy compared to the individual treatments of BoNT/A or PRP alone (*P* < 0.01). Subsequently, immunofluorescence analysis was employed to evaluate the expression levels of key molecules in the Wnt signaling pathway, namely β-catenin and the extracellular matrix protein versican (Fig. 1G-1J). The results demonstrated that the BoNT/A and PRP groups exhibited significantly elevated expression levels of both β-catenin and versican proteins compared to the Control group (*P* < 0.01). Moreover, compared with the individual treatments of BoNT/A and PRP, the combination treatment (BoNT/A+PRP) further enhanced the expression of these proteins (*P* < 0.01). These results substantiated that BoNT/A and PRP effectively promoted hair follicle growth, with the combination treatment of both agents yielding superior efficacy compared to their individual treatments.

### BoNT/A and PRP Treatment Upregulates the Expression of STMN1 Protein in DPCs

**Fig. 2:**
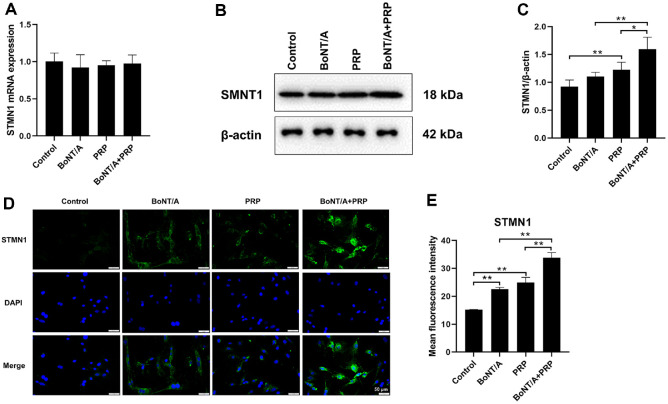
BoNT/A and PRP treatment upregulated the expression of STMN1 protein. (**A**) The expression level of the STMN1 gene was detected using qRT-PCR. (**B-C**) Western blot detection of STMN1. (**D-E**) Immunofluorescence staining was used to detect the expression levels of STMN1 (20X). *P < 0.05, **P < 0.01.

STMN1 gene expression did not differ significantly across the four groups (*P* > 0.05, Fig. 2A). However, the PRP group exhibited a significant increase in STMN1 protein expression compared to the Control group (*P* < 0.05, Fig. 2B and 2C). β-actin served as the loading control for all Western blots; representative β-actin bands are shown in Fig. 2B and 2C. Moreover, the BoNT/A+PRP group demonstrated significantly higher STMN1 protein expression compared to either the BoNT/A or PRP groups alone (*P* < 0.05, *P* < 0.01). The immunofluorescence staining demonstrated that both BoNT/A and PRP significantly upregulated STMN1 protein expression (*P* < 0.01), with the combination treatment of BoNT/A and PRP exhibiting the most pronounced promoting effect on STMN1 protein expression (*P* < 0.01, Fig. 2D and 2E). These data indicated that the combined treatment of BoNT/A and PRP upregulated the expression of STMN1 protein.

### BoNT/A and PRP Promote Hair Follicle Growth by Up-Regulating STMN1 Protein and Activating the Wnt/β-Catenin Signaling Pathway

**Fig. 3:**
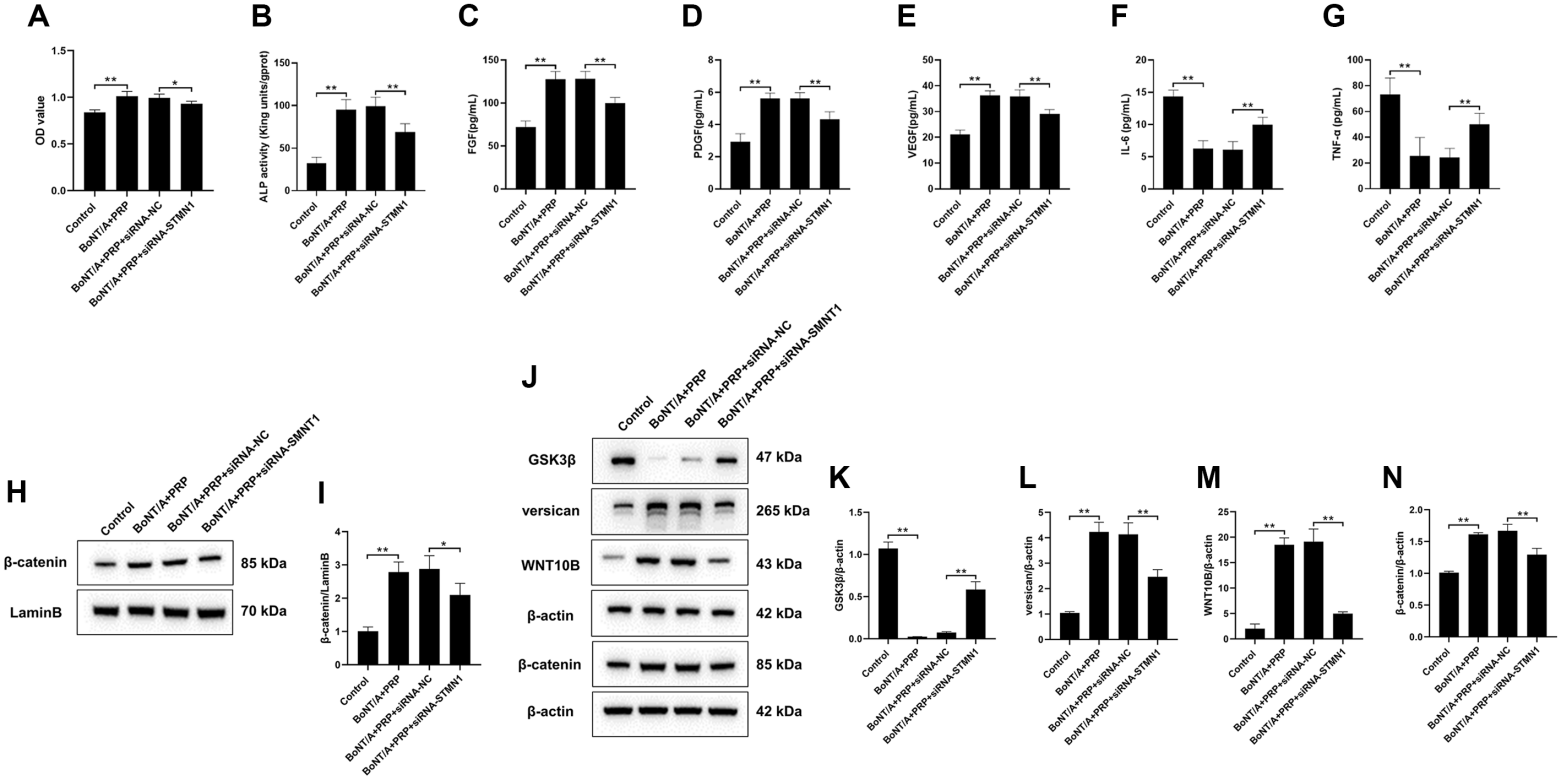
BoNT/A and PRP promote hair follicle growth by up-regulating STMN1 protein and activating the Wnt/β-catenin signaling pathway. (**A-G**) The levels of ALP, FGF, PDGF, VEGF, IL-6, and TNF-α in DPCs. (**H-N**) Western blot detection of β-catenin in the cell nucleus and GSK3β, versican, WNT10B, and β-catenin in the DPCs. The data are expressed as the mean ± SD. *P < 0.05, **P < 0.01.

First, the CCK-8 assay was employed to evaluate the viability of DPCs (Fig. 3A). The results demonstrated that, compared with the Control group, cell viability in the BoNT/A+PRP group was significantly increased (*P*<0.01). In contrast, cell viability in the BoNT/A+PRP+siRNA-STMN1 group was markedly reduced compared with the BoNT/A+PRP+siRNA-NC group (*P* < 0.05). Subsequently, the levels of hair follicle-related growth factors were examined (Fig. 3B-3G). The results demonstrated that BoNT/A and PRP combination therapy markedly increased ALP, FGF, PDGF, and VEGF expression while suppressing IL-6 and TNF-α levels relative to controls (*P* < 0.01). Notably, silencing STMN1 reversed the promoting effect of the combined treatment on hair follicle growth (*P* < 0.01). Western blot analysis further showed that the BoNT/A+PRP group significantly enhanced the nuclear expression of β-catenin protein in DPCs, as well as the cellular expression of β-catenin, WNT10B, and versican proteins, while reducing the expression of GSK3β protein (*P* < 0.01). Silencing STMN1 reversed these protein expression changes (*P* < 0.01, Fig. 3H-3N). Collectively, these data indicated that the combined treatment of BoNT/A and PRP activated the Wnt/β-catenin signaling pathway via upregulation of STMN1 protein, thereby promoting hair follicle growth.

### Hair Follicle Growth Records of BALB/C Nude Mice

**Fig. 4:**
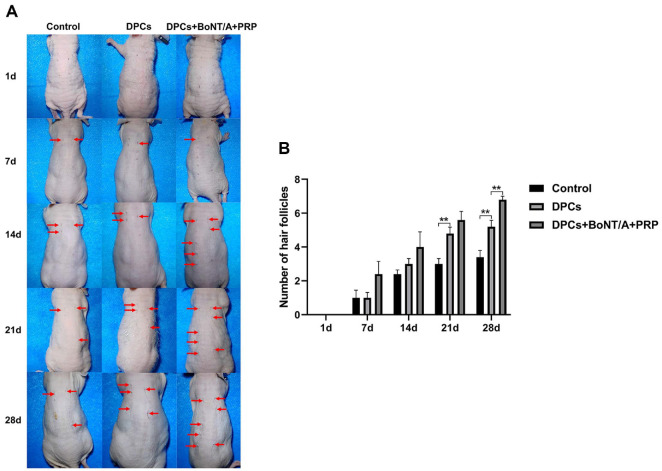
Hair follicle growth records of BALB/C nude mice. (**A-B**) The number of hair follicles in BALB/C nude mice (1 day, 7 days, 14 days, 21 days, 28 days post-operation). The data are expressed as the mean ± SD. **P < 0.01.

The growth of hair follicles was monitored weekly post-surgery, with photographic documentation and quantification of follicle numbers. This observation period spanned 28 days. As depicted in Figure 4A and 4B, the number of hair follicles progressively increased over time in all groups. At 21 and 28 days, the DPCs group exhibited a significantly higher number of hair follicles compared to the control group. Notably, at 28 days, mice treated with the combination of BoNT/A and PRP demonstrated a significantly greater number of hair follicles than those in the DPCs group. These findings suggest that the combined treatment of BoNT/A and PRP effectively promotes hair follicle growth in mice.

### *In Vivo* Verification of the Combined Promotion of Hair Follicle Growth by BoNT/A and PRP

**Fig. 5:**
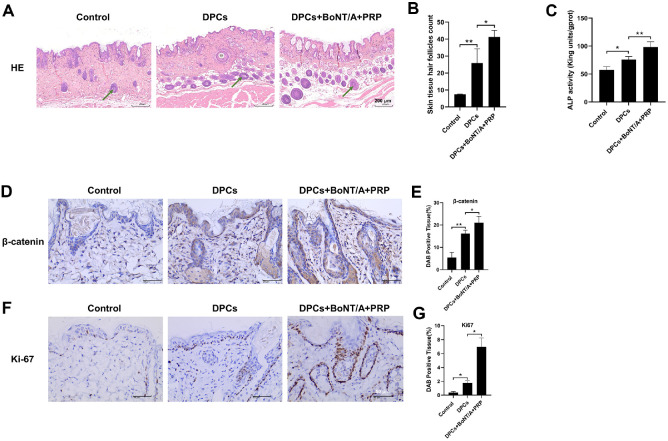
In vivo verification of the combined promotion of hair follicle growth by BoNT/A and PRP. (**A-B**) HE staining was performed for hair follicle counting (×100). Green arrow: hair follicle. (**C**) The levels of ALP in the dorsal skin containing hair follicles. (**D-G**) Immunohistochemical staining was performed to detect the expression of β-catenin and Ki-67 (40X). The data are expressed as the mean ± SD. *P < 0.05, **P < 0.01.

The dorsal skin containing hair follicles was dissected and subjected to HE staining for quantification of hair follicle density (Fig. 5A). The DPCs group showed a greater number of hair follicles in skin tissue than the Control group (*P* < 0.01). Moreover, the DPCs+BoNT/A+PRP group showed a significantly higher number of hair follicles in the skin tissue compared to the DPCs group (*P* < 0.05). As shown in Fig. 5B, ELISA analysis of the skin containing hair follicles revealed that the ALP level in the DPCs group was significantly higher than that in the Control group (*P* < 0.05). Moreover, the ALP level in the DPCs+BoNT/A+PRP group was further elevated compared to the DPCs group (*P* < 0.01). IHC staining revealed that DPCs significantly promoted the expression of β-catenin and Ki67 (*P* <0.05, *P* < 0.01). Moreover, the combined treatment of BoNT/A and PRP exhibited a more pronounced effect on enhancing the expression of β-catenin and Ki67 compared to the DPCs group (*P* < 0.05). The aforementioned data demonstrated that the combination of BoNT/A and PRP effectively promoted *in vivo* hair follicle growth.

## Discussion

AGA, the most prevalent form of hair loss encountered in clinical practice, is driven by specific genetic predispositions and androgenic influences, causing hair follicles to undergo progressive miniaturization and hair loss to exhibit a characteristic distribution pattern where it predominantly affects the frontal hairline and temporal regions in men and more frequently involves the vertex scalp in women [23]. The limited efficacy of conventional treatment regimens has necessitated the ongoing exploration of novel therapeutic strategies. This study focuses on the treatment of AGA using BoNT/A in combination with PRP, exploring the mechanism by which this approach promotes hair follicle growth and reconstruction through the regulation of the STMN1 protein and activation of the Wnt/β-catenin signaling pathway.

BoNT/A is a highly potent neurotoxin. Several studies have highlighted its efficacy in treating AGA. Shon *et al*. administered BoNT/A via intradermal injection to 49 AGA patients with an average age of 30 years, and the results demonstrated a significant improvement in hair density [18]. Singh *et al*. further confirmed the therapeutic potential of BoNT/A through intramuscular injection for AGA treatment [24]. PRP mitigates hair follicle apoptosis and enhances cellular proliferation by releasing substantial amounts of PDGF, FGF, VEGF, and other growth factors into the microenvironment, consequently promoting hair growth and preserving hair follicle integrity [25]. Starace *et al*. demonstrated that PRP as an adjunctive treatment for female AGA, it was concluded that PRP positively impacts both hair density and hair diameter [26]. According to Butt *et al*., their research on PRP treatment for AGA demonstrated that an increase in hair density after 6 months of treatment suggests that PRP is a viable and effective therapeutic option for managing AGA [27]. However, studies have demonstrated that the combination of PRP and pharmacological therapy yields superior therapeutic outcomes compared to single-modality treatments [28, 29]. Versican, serving as a marker for mature dermal papilla cells, plays a critical role in regulating the adhesion, proliferation, migration, and differentiation of Dermal papilla (DP) cells and is essential for maintaining and inducing the anagen phase of hair growth [30]. The marked reduction of versican expression in dermal papilla cells from both androgenetic alopecia and vellus-like hair follicles suggests that versican is indispensable for promoting and sustaining hair growth in animal models [31]. In this study, it was observed that both BoNT/A and PRP, when applied individually, as well as their combined application, significantly promoted the upregulation of ALP, FGF, PDGF, VEGF, and versican. Notably, the synergistic effect of BoNT/A and PRP was superior to either treatment alone, aligning with findings from prior studies. In the animal hair follicle transplantation model, results consistent with *in vitro* cell experiments were observed. The combined treatment of BoNT/A and PRP led to an increase in hair follicle number, upregulation of ALP levels, and enhanced expression of β-catenin and Ki-67. These results suggest that the combination of BoNT/A and PRP effectively enhances hair follicle growth.

The hair cycle is regulated by multiple signaling pathways, including SHH, Wnt, and Notch. The Wnt/β-catenin signaling pathway plays a central role in hair morphogenesis, cycling, and follicle regeneration during both embryonic development and adulthood. The WNT/β-catenin signaling pathway functions as a critical switch for hair follicle development. In skin with either absent β-catenin expression or elevated expression of the WNT inhibitor DKK1, hair follicle induction is impaired. DPCs treated with WNT-3A or WNT-10B exhibit significantly enhanced capacity for hair formation compared to untreated controls [32]. Furthermore, the maintenance and growth of hair follicles depend on interactions within the Wnt signaling network. For example, WNT3A and WNT10B promote cell proliferation and differentiation, facilitating the transition of hair follicles from the telogen phase to the anagen phase during the hair cycle [20]. Evidence suggests that Wnt signaling is implicated in the pathogenesis of AGA and is directly modulated by androgens. In a recent study, differentially expressed mRNAs in AGA were found to be predominantly enriched in the Wnt signaling pathway [33]. Glycogen synthase kinase-3β (GSK-3β), which is activated by androgens, is an enzyme responsible for the phosphorylation and ubiquitination-mediated degradation of β-catenin. However, the binding of dihydrotestosterone (DHT) to the androgen receptor (AR) inhibits the dephosphorylation of GSK-3β, leading to the promotion of Wnt/β-catenin signaling degradation [34]. This disruption contributes to hair cycle disorders observed in AGA. It is evident from these findings that the Wnt signaling pathway critically disrupts the hair growth cycle in AGA.

Although AGA has traditionally been considered a non-inflammatory form of hair loss, multiple studies have demonstrated that a certain level of inflammation exists in the scalps of some AGA patients. This type of inflammation is subtle and progresses slowly without overt inflammatory signs, leading to its characterization as "microinflammation." In addition to hair follicle miniaturization and cycle disorders, microinflammation represents another key feature of AGA [35]. Plante *et al*. demonstrated that the severity of AGA and the extent of hair follicle miniaturization were significantly associated with the degree of inflammatory infiltration [36]. Moreover, pronounced inflammatory infiltration was observed surrounding highly miniaturized hair follicles. Miao *et al*. performed a comprehensive transcriptome analysis across different scalp regions in AGA patients and discovered that inflammatory genes, such as TNF-α and IL-6, were consistently upregulated compared to normal levels in all AGA groups[37]. The results of this study demonstrated that the combination of BoNT/A and PRP significantly decreased IL-6 and TNF-α levels, with a more pronounced effect compared to either treatment used alone. By mitigating inflammation, this approach may potentially inhibit hair follicle miniaturization.

Stathmin 1 (STMN1) is a protein that destabilizes microtubules, thereby playing a critical role in mitosis and cellular migration by regulating the availability of free tubulin dimers within cells [38]. Depletion of STMN1 triggers cell cycle arrest and promotes apoptotic cell death [39, 40]. Research has demonstrated that the microtubule-associated protein STMN1 activates Wnt/β-Catenin signaling, thereby promoting osteogenic differentiation [41]. Furthermore, Bichsel *et al*. demonstrated that deletion of STMN1 accelerates the transition to catagen and prematurely suppresses follicular proliferation, indicating its essential role in hair follicle cycling [42]. Additionally, further study has confirmed that STMN1 serves as a critical factor in facilitating hair follicle regeneration [43]. In the context of hair follicle regeneration, STMN1 plays a pivotal role. Research indicated that STMN1 facilitates hair follicle cell proliferation and regeneration by modulating the Wnt/β-Catenin signaling pathway [44]. Consistent with previous reports, STMN1 protein is detected in DPCs, and the combined application of BoNT/A and PRP upregulates STMN1 expression. Upon silencing STMN1, we observed that it abrogates the synergistic effects of BoNT/A and PRP, thereby inhibiting hair follicle growth. Our research findings demonstrated that the combined application of BoNT/A and PRP promotes hair follicle growth by activating the Wnt/β-catenin signaling pathway through STMN1 upregulation.

This study has certain inherent limitations. Transient siRNA knockdown sufficed to establish STMN1 as a requisite mediator of BoNT/A+PRP-driven hair-follicle growth, yet the intervention was acute; validation in organoid or long-term culture systems would clarify durability. Similarly, preliminary animal numbers and the 6-month clinical follow-up were modest; expanded cohorts and extended surveillance are warranted to consolidate efficacy and safety profiles.

## Conclusion

In conclusion, our findings demonstrate that the combination of BoNT/A and PRP effectively increases hair follicle density, promotes hair follicle growth, and potentially inhibits hair follicle miniaturization by mitigating inflammatory responses. The therapeutic effect arises from Wnt/β-catenin pathway activation driven by enhanced STMN1 expression. We anticipate that our results will offer novel insights and strategies for the treatment of AGA. Nevertheless, further investigations are warranted to evaluate the long-term efficacy and safety profile of this combined therapy.

## Figures and Tables

**Table 1 T1:** Primers sequence.

Primer	Forward primer (5’→3’)	Reverse primer (5’→3’)
STMN1	aaagaagaaggacctttccctg	gaagttgttgttctcctcgatg
β-actin	ctacctcatgaagatcctgacc	cacagcttctctttgatgtcac
